# Dominant aminoacyl-tRNA synthetase disorders: lessons learned from *in vivo* disease models

**DOI:** 10.3389/fnins.2023.1182845

**Published:** 2023-05-12

**Authors:** Elizabeth Kalotay, Matthias Klugmann, Gary D. Housley, Dominik Fröhlich

**Affiliations:** ^1^Translational Neuroscience Facility and Department of Physiology, School of Biomedical Sciences, University of New South Wales, Sydney, NSW, Australia; ^2^Research Beyond Borders, Boehringer Ingelheim Pharma GmbH & Co. KG, Biberach an der Riss, Germany

**Keywords:** aminoacyl-tRNA synthetases, ARS1, dominant mutations, peripheral neuropathy, Charcot-Marie-Tooth disease, CMT, animal models

## Abstract

Aminoacyl-tRNA synthetases (ARSs) play an essential role in protein synthesis, being responsible for ligating tRNA molecules to their corresponding amino acids in a reaction known as ‘tRNA aminoacylation’. Separate ARSs carry out the aminoacylation reaction in the cytosol and in mitochondria, and mutations in almost all ARS genes cause pathophysiology most evident in the nervous system. Dominant mutations in multiple cytosolic ARSs have been linked to forms of peripheral neuropathy including Charcot-Marie-Tooth disease, distal hereditary motor neuropathy, and spinal muscular atrophy. This review provides an overview of approaches that have been employed to model each of these diseases *in vivo*, followed by a discussion of the existing animal models of dominant ARS disorders and key mechanistic insights that they have provided. In summary, ARS disease models have demonstrated that loss of canonical ARS function alone cannot fully account for the observed disease phenotypes, and that pathogenic ARS variants cause developmental defects within the peripheral nervous system, despite a typically later onset of disease in humans. In addition, aberrant interactions between mutant ARSs and other proteins have been shown to contribute to the disease phenotypes. These findings provide a strong foundation for future research into this group of diseases, providing methodological guidance for studies on ARS disorders that currently lack *in vivo* models, as well as identifying candidate therapeutic targets.

## 1. Introduction

Aminoacyl-tRNA synthetases (ARSs) are a group of enzymes that pair amino acids to their complementary tRNA molecules (tRNA aminoacylation), facilitating their incorporation into newly synthesized proteins. tRNA aminoacylation is performed for each proteinogenic amino acid by a specific ARS, and this process occurs separately within the cytosol and mitochondria of each cell (Fröhlich et al., 2018). In the first step of the aminoacylation reaction, known as amino acid ‘activation’, the ARS binds its corresponding amino acid and hydrolyses an ATP molecule to produce an aminoacyl-adenylate (aa-AMP). In the second step of the reaction, this activated amino acid residue is ligated to a matching tRNA molecule, and the AMP molecule is released.

Misacylation of tRNA molecules can lead to the misincorporation of amino acids during protein synthesis, which can be highly deleterious to cellular function (Nangle et al., 2006; Fröhlich et al., 2017; Kapur and Ackerman, 2018). To maintain high fidelity of protein translation, if an ARS incorrectly activates the wrong amino acid, this error can be rectified through hydrolysis of the aa-AMP prior to the second step of the aminoacylation reaction (‘pre-transfer editing’), or after the tRNA molecule has been misacylated (‘post-transfer editing’). Multiple ARSs possess editing domains for post-transfer editing, and editing can also be carried out *in trans* by separate editing proteins (Perona and Gruic-Sovulj, 2014).

Genes encoding ARSs are named according to their target amino acid, and whether they function within the cytosol (*ARS1*) or mitochondria (*ARS2*); for example, the cytosolic tyrosyl-tRNA synthetase (TyrRS) is encoded by *YARS1*, and its mitochondrial counterpart is encoded by *YARS2*. In most cases, cytosolic and mitochondrial ARSs are encoded by separate genes, apart from two bifunctional ARSs; glycyl-tRNA synthetase (GlyRS) and lysyl-tRNA synthetase (LysRS), which are each encoded by a single gene. All ARS genes are encoded by nuclear DNA and translated in the cytosol, with mitochondrial ARSs subsequently imported into the mitochondria.

In addition to their canonical role in tRNA aminoacylation, ARSs have evolved to perform several additional cellular functions. These secondary ARS functions have been reviewed elsewhere (Guo and Schimmel, 2013; Lo et al., 2014), with some examples of non-canonical ARS functions including regulation of vascular development (Mirando et al., 2014), immune responses (Nie et al., 2019), and metabolism (Arif et al., 2017).

Mutations in all ARS genes have been linked to disease. Recessive mutations in cytosolic and mitochondrial ARS genes often cause multi-system disorders, predominantly involving the central nervous system (Ognjenovic and Simonovic, 2018; Fuchs et al., 2019; Muthiah et al., 2020). To date, dominant mutations in ARS2 genes have not been implicated in human disease. In contrast, dominant mutations in multiple cytosolic and bifunctional ARS genes have been linked to peripheral neuropathies (Wei et al., 2019). Recently, the clinical spectrum associated with monoallelic ARS1 variants has been expanded to also include disorders of the central nervous system, which resemble diseases typically associated with recessive ARS1 mutations. Specifically, mutations in *AARS1* have been identified as the likely cause of Swedish-type hereditary diffuse leukoencephalopathy with spheroids 2 (HDLS2 or HDLS-S) (Sundal et al., 2019), and a dominant *HARS1* variant has been linked to cerebellar degeneration, cognitive impairments, and peripheral neuropathy (Royer-Bertrand et al., 2019). Dominant mutations in *NARS1* have been identified as a cause of neurodevelopmental disorder with microcephaly, impaired language, epilepsy, and gait abnormalities (NEDMILEG) (Manole et al., 2020). Additionally, a monoallelic *KARS1* variant was recently linked to a case of severe anaphylaxis resulting from dysregulation of the non-canonical function of LysRS in activating immune cells (Ribó et al., 2021). The differing inheritance pattern and largely non-overlapping clinical presentations of patients with central and peripheral nervous system disorders suggests that these represent distinct groups of diseases, with divergent pathomechanisms. This review will primarily focus on the association between dominant ARS1 variants and peripheral neuropathy.

## 2. Peripheral neuropathies caused by dominant mutations in cytosolic ARSs

Thus far, autosomal dominant mutations in *AARS1*, *GARS1, HARS1, MARS1, WARS1, SARS1* and *YARS1* have been linked to peripheral neuropathies, predominantly Charcot-Marie-Tooth (CMT) disease (Table 1). CMT is characterized by length-dependent degeneration of peripheral motor and sensory neurons, causing distal muscle atrophy and weakness, sensory loss, and diminished deep tendon reflexes (Gutmann and Shy, 2015). Neuronal degeneration can result from demyelination (CMT Type 1; CMT1), or from primary axonal dysfunction (CMT Type 2; CMT2). These categories can be differentiated electrophysiologically, with motor nerve conduction velocities (MNCV) <38 m/s indicative of CMT1, and MNCV >38 m/s designated as CMT2. Individuals with MNCV between 25 m/s and 45 m/s are generally classified as having an intermediate form of CMT (Reilly, 2009). ARS1 mutations have so far been associated with CMT2 and dominant intermediate CMT (DI-CMT), as well as forms of distal hereditary motor neuropathy (dHMN) and spinal muscular atrophy (SMA), which have overlapping clinical features with CMT. There are currently no curative treatments for ARS-associated neuropathies, or for inherited peripheral neuropathies in general.

**Table 1:** Diseases caused by dominant mutations in ARS genes and associated animal studies.

Protein (Gene)	Disease(s)	Animal studies and disease models
Alanyl-tRNA synthetase (***AARS1***)	Charcot-Marie-Tooth disease (Axonal, type 2N (CMT2N) [OMIM #613287] and Dominant Intermediate CMT (DI-CMT)) (Latour et al., 2010; McLaughlin et al., 2012; Bansagi et al., 2015; Weterman et al., 2018; Hsu et al., 2019; Imaizumi et al., 2019; Tatsumi et al., 2019; Lee et al., 2020; Sun et al., 2021; Høyer et al., 2022) CMT2N with posterior reversible encephalopathy syndrome (Huang H. et al., 2022) Myeloneuropathy (Motley et al., 2015; Sun et al., 2021) Distal hereditary motor neuropathy (dHMN) (Zhao et al., 2012; Yuan et al., 2022) Hereditary diffuse leukoencephalopathy with spheroids 2 (HDLS2) [OMIM #619661] (Sundal et al., 2019)	**Zebrafish** (***Danio rerio***) Expression of CMT2N patient variants (p.R326W, p.E337K, p.S627L) (Weterman et al., 2018)
Glycyl-tRNA synthetase (***GARS1***)	Charcot-Marie-Tooth disease, type 2D (CMT2D) [OMIM #601472] (Antonellis et al., 2003; Sivakumar et al., 2005; Del Bo et al., 2006; James et al., 2006; Cader et al., 2007; Nangle et al., 2007; Xie et al., 2007; Abe and Hayasaka, 2009; Hamaguchi et al., 2010; He et al., 2011; Eskuri et al., 2012; Griffin et al., 2014; Kawakami et al., 2014; He et al., 2015; Liao et al., 2015; Sun et al., 2015; Mo et al., 2018; Yalcouyé et al., 2019; Meyer et al., 2022) Distal hereditary motor neuronopathy, type VA (dHMN5A) [OMIM #600794] (Antonellis et al., 2003; Sivakumar et al., 2005; Antonellis et al., 2006; Del Bo et al., 2006; Dubourg et al., 2006; James et al., 2006; Nangle et al., 2007; Rohkamm et al., 2007; He et al., 2011; Lee et al., 2012; Griffin et al., 2014; Sleigh et al., 2017b; Chung et al., 2018; Mo et al., 2018; Yu et al., 2018; Lee et al., 2019; Huang Y. et al., 2022) Infantile spinal muscular atrophy, James type (SMAJI) [OMIM #619042] (James et al., 2006; He et al., 2011; Eskuri et al., 2012; Griffin et al., 2014; Mo et al., 2018; Markovitz et al., 2020; Meyer et al., 2022) Typical bulbar ALS (Corcia et al., 2019) DI-CMT (Nan et al., 2019)	***Drosophila melanogaster*** Ubiquitous and conditional overexpression of p.G240R and p.P234KY *GlyRS* variants (Ermanoska et al., 2014; Grice et al., 2015, 2018) Expression of human p.E71G, p.G240R and p.G526R *GARS1* variants (Niehues et al., 2015) SIRT2 knockdown in *Drosophila* expressing human p.G526R variant (Zhao et al., 2021) tRNA^Gly^ overexpression in *Drosophila* expressing human p.G240R, p.G526R and p.E71G variants (Zuko et al., 2021) Introduction of p.P98L mutation into *GlyRS* (identified through forward genetic screen looking at dendritic and axonal development) (Chihara et al., 2007) Overexpression of semaphorin-2a to restore semaphorin signaling in *GlyRS^P234KY^ Drosophila* (Grice et al., 2018) SIRT2 inhibition in *GlyRS^G256R^ Drosophila* (Zhao et al., 2021) **Zebrafish** (***Danio rerio***) ‘s266’ *gars1* mutation (p.T209K; equivalent to p.T130K in human GlyRS) identified in an ENU screen (Malissovas et al., 2016) Overexpression of zebrafish *gars1* p.236R (p.C157R in human GlyRS), *gars1* p.G319R (p.G240R in human GlyRS), and *gars1* p.G605R (p.G526R in human GlyRS) in wildtype, heterozygous and homozygous s266 zebrafish embryos (Malissovas et al., 2016) **Mouse** (***Mus musculus***) ‘Nmf249’ (*Gars1* p.P278KY; human equivalent p.234KY) variant produced through mutagenesis (Seburn et al., 2006; He et al., 2015; Bais et al., 2016; Spaulding et al., 2016; Mo et al., 2018; Morelli et al., 2019; Ozes et al., 2021; Spaulding et al., 2021) Loss-of-function allele (gene trap XM256 insertion in intron 2) (Seburn et al., 2006) *Gars1* p.C201R (human equivalent p.C157R) variant produced through mutagenesis (Achilli et al., 2009; Bais et al., 2016; Spaulding et al., 2016, 2021; Sleigh et al., 2017b, 2020a,b; Benoy et al., 2018; Boczonadi et al., 2018; Mo et al., 2018; Zuko et al., 2021) CRISPR/Cas9-mediated introduction of ΔETAQ (CMT2D patient) mutation into mouse *Gars1* gene (Morelli et al., 2019; Ozes et al., 2021; Spaulding et al., 2021; Zuko et al., 2021) Adenovirus-mediated expression of wildtype, p.L129P and p.G240R human GlyRS variants in spinal cord and DRG neurons (Lee et al., 2014; Seo et al., 2014a,b) VEGF-A administration to *Gars1^P278KY/+^* mice (He et al., 2015) Intraperitoneal administration of physostigmine to *Gars1^P278KY/+^* and *Gars1^C201R/+^* mice (Spaulding et al., 2016) GCN2 deletion and inhibition in *Gars1^P278KY/+^* and *Gars1^delETAQ/+^* mice (Spaulding et al., 2021) AAV1.NT-3 treatment of *Gars1^P278KY/+^* and *Gars1^delETAQ/huEx8^* mice (Ozes et al., 2021) AAV-mediated RNAi of *Gars1* p.P278KY and ΔETAQ variants (Morelli et al., 2019) HDAC6 inhibition in *Gars1^C201R/+^* and *Gars1^P278KY/+^* mice (Benoy et al., 2018)
Histidyl-tRNA synthetase (***HARS1***)	Axonal Charcot-Marie-Tooth disease, type 2W (CMT2W) [OMIM #616625] (Vester et al., 2013; Safka Brozkova et al., 2015; Abbott et al., 2018) Late-onset demyelinating peripheral neuropathy, cerebellar atrophy, and cognitive deficit (Royer-Bertrand et al., 2019)	***C. elegans*** *C. elegans hars-1* p.Arg137Gln expressed in GABA motor neurons (Vester et al., 2013) *C. elegans hars-1* p.Asp364Tyr expressed in GABA motor neurons (Safka Brozkova et al., 2015) **Zebrafish** (***Danio rerio***) Expression of human p.V155G, p.Y330C and p.R137Q HisRS variants (Mullen et al., 2021)
Lysyl-tRNA synthetase (***KARS1***)	Dominant mutation found to cause anaphylaxis by interfering with LysRS secondary function in mast cell activation (Ribó et al., 2021)	None
Methionyl-tRNA synthetase (***MARS1***)	Axonal Charcot-Marie-Tooth disease, type 2U (CMT2U) [OMIM #616280] (Gonzalez et al., 2013; Hyun et al., 2014; Hirano et al., 2016; Nam et al., 2016; Sagi-Dain et al., 2018; Gillespie et al., 2019)	None
Asparaginyl-tRNA synthetase (***NARS1***)	Neurodevelopmental Disorder with Microcephaly, Impaired Language, Epilepsy and Gait Abnormalities, autosomal dominant; NEDMILEG [OMIM #619092] (Manole et al., 2020)	**Zebrafish** (***Danio rerio***) Expression of the human p.Arg534* AsnRS variant that causes NEDMILEG through microinjection of human *NARS1* RNA into zebrafish embryos (Manole et al., 2020)
Seryl-tRNA synthetase (***SARS1***)	Charcot-Marie-Tooth disease (He et al., 2022)	None
Tryptophanyl-tRNA synthetase (***WARS1***)	Distal Hereditary Motor Neuronopathy, type IX (dHMN9) [OMIM #617721] (Tsai et al., 2017; Wang et al., 2019)	None
Tyrosyl-tRNA synthetase (***YARS1***)	Dominant intermediate Charcot-Marie-Tooth disease, type C (DI-CMTC) [OMIM #608323] (Jordanova et al., 2006; Hyun et al., 2014; Gonzaga-Jauregui et al., 2015; Thomas et al., 2016)	***Drosophila melanogaster*** Expression of DI-CMTC patient p.E196K variant (Storkebaum et al., 2009; Ermanoska et al., 2014; Niehues et al., 2015; Bervoets et al., 2019) Expression of p.G41R patient variant (Storkebaum et al., 2009; Niehues et al., 2015) Expression of p.Δ153-156VKQV patient variant (Storkebaum et al., 2009; Niehues et al., 2015) Expression of benign p.K256N variant in *TyrRS* used to assess utility of *Drosophila* for screening variant pathogenicity (Leitão-Gonçalves et al., 2012) Blocking nuclear localization of TyrRS in *YARS1^E196K^* expressing *Drosophila* (Bervoets et al., 2019) **Mouse** (***Mus musculus***) Introduction of p.E196K mutation into *Yars1* (Hines et al., 2021; Spaulding et al., 2021) Gene trap allele ‘RRC100’ (in exon 11 of *Yars1*) expressed alone or in combination with p.E196K mutation (Hines et al., 2021) Adenovirus-mediated expression of wildtype, p.E196K and p.G41R human *YARS1* variants (Lee et al., 2017)

**Figure 1:**
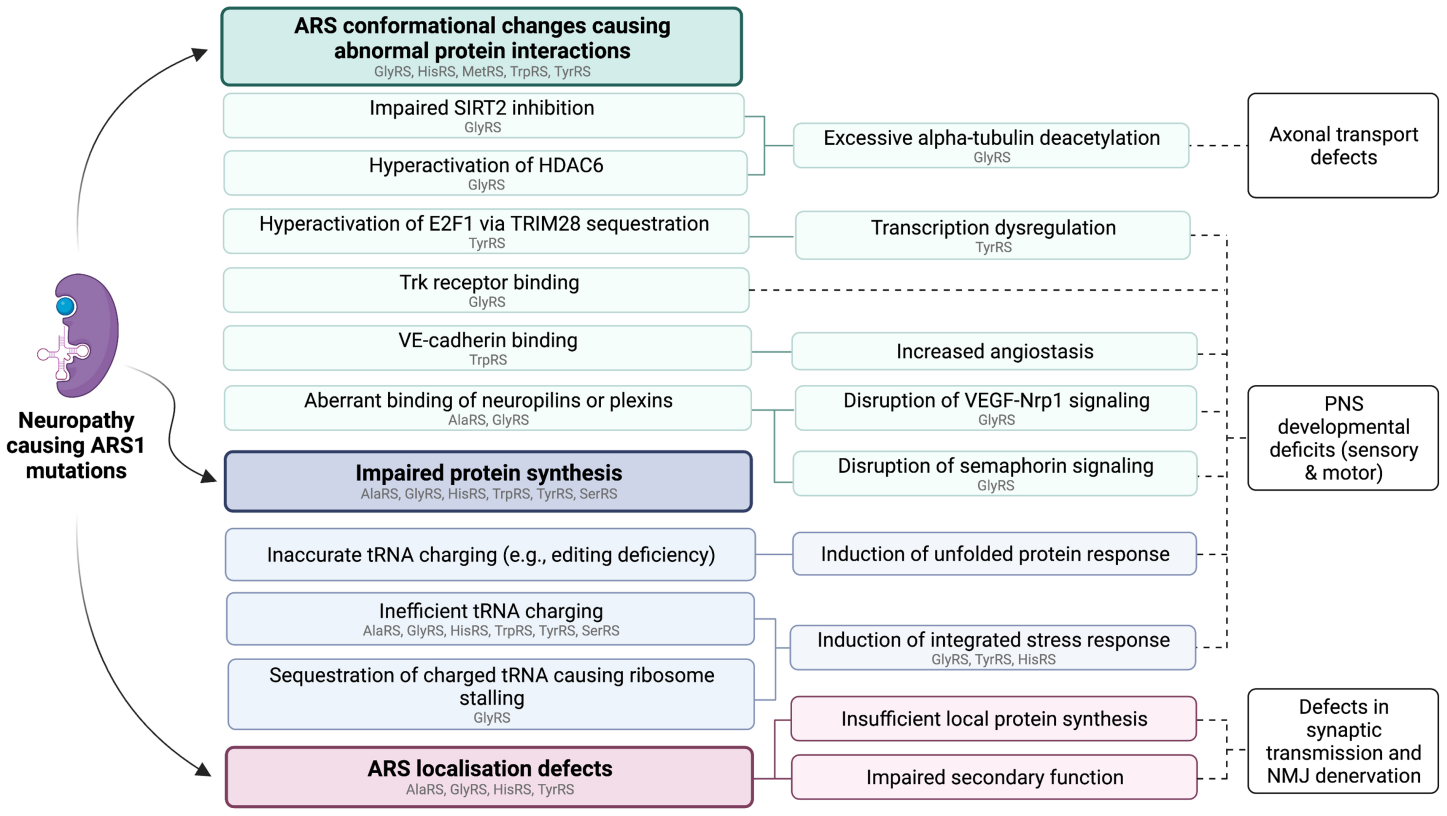
Summary of proposed functional consequences of dominant ARS1 mutations. These pathways are not mutually exclusive and may act synergistically to cause ARS1-associated neuropathy. Individual variants of the same ARS may cause disease via different mechanisms. ARSs with variants that have so far been implicated in specific pathways through *in vitro* or *in vivo* studies are listed in gray. This figure was created using BioRender.com.

Based on the available evidence, loss of aminoacylation function cannot fully account for the association between dominant ARS mutations and neuropathy. For neuropathy associated ARS1 variants, loss of aminoacylation function *in vitro* is a common feature, but not a prerequisite for disease (Nangle et al., 2007). Additionally, heterozygous knockouts of ARS1 genes implicated in dominant human ARS diseases are not sufficient to trigger the disease phenotypes in mice (Seburn et al., 2006; Dickinson et al., 2016; Hines et al., 2021), and human heterozygous carriers of loss-of-function ARS mutations are not always affected (Galatolo et al., 2020; Kuo et al., 2020). This indicates that the neuropathy phenotype is not caused by haploinsufficiency, suggesting that neuropathy-linked ARS variants exert dominant-negative effects on protein function or confer toxic gain-of-function effects. An overview of proposed disease pathways for ARS-associated neuropathies is provided in Figure 1.

*In vivo* models for neuropathy causing ARS1 variants have been produced in *Caenorhabditis (C.) elegans*, *Drosophila melanogaster*, zebrafish, and mice (Table 1). The dominant neurotoxicity of two CMT-causing *HARS1* variants have been evaluated through overexpression of equivalent variants in *C. elegans* (Vester et al., 2013; Safka Brozkova et al., 2015). In *Drosophila*, RNA interference (RNAi)-mediated ARS1 knockdown and expression of multiple ARS1 variants have been investigated using the UAS-GAL4 system (Morant et al., 2021). Zebrafish CMT models have been created using forward genetic screens, expression of hypomorphic and hypermorphic human ARS1 variants (Weterman et al., 2018), and overexpression of mutant ARSs equivalent to human patient mutations (Malissovas et al., 2016; Mullen et al., 2021). Mouse models for CMT have been generated using forward genetic screens (Seburn et al., 2006; Achilli et al., 2009), gene trapping (Seburn et al., 2006), and through the introduction of human disease-causing mutations into the mouse genome (Morelli et al., 2019).

Models for ARS1-associated neuropathy have so far been generated for *GARS1, YARS1, HARS1,* and *AARS1*. There are currently no animal studies addressing dHMN9 caused by *WARS1* mutations, CMT2U caused by *MARS1* mutations, or for the form of CMT caused by *SARS1* mutations.

### 2.1. Dominant *GARS1* mutations and disease

Dominant mutations in *GARS1*, which encodes the bifunctional glycyl-tRNA synthetase (GlyRS), cause CMT2D and distal spinal muscular atrophy type V (DSMAV) (Antonellis et al., 2003). CMT2D and DSMAV patients typically present with slowly progressing muscle weakness and atrophy, most pronounced in the upper extremities. In addition to motor impairment, CMT2D patients exhibit sensory loss. The severity of sensory deficits among CMT2D patients is variable, predominantly affecting the upper extremities. *GARS1* mutations have also been linked to a form of infantile spinal muscular atrophy with a typical clinical presentation of progressive muscle weakness in both upper and lower limbs, sometimes preventing independent ambulation (James et al., 2006; Markovitz et al., 2020). Additionally, some patients present with respiratory failure and eating difficulties (Markovitz et al., 2020). For each of these *GARS1*-associated disorders, patients exhibit variable reductions in MNCV, as well as in sensory nerve action potential (SNAP) and compound motor action potential (CMAP) amplitudes. Sural nerve biopsies show evidence of axonal degeneration, without signs of demyelination (Sivakumar et al., 2005).

Despite the different disease classifications, there is substantial phenotypic overlap between the *GARS1*-associated neuropathies. DSMAV and CMT2D can be caused by the same *GARS1* mutations (Sivakumar et al., 2005; Del Bo et al., 2006), suggesting that these conditions represent a spectrum of *GARS1* associated axonal neuropathy, rather than distinct diseases (Sivakumar et al., 2005; Sleigh et al., 2017a).

#### 2.1.1. Mouse models of CMT2D

As the first ARS to be associated with human disease, GlyRS mutations have been the most extensively studied, and were the first dominant ARS variants to be modeled in mammals.

The first mouse model of CMT2D was established in 2006 after identification of the dominant *Nmf249 Gars1* mutation (P278KY; equivalent to human P234KY) in a forward genetic screen (Seburn et al., 2006). The P278KY amino acid substitution is located near the human disease-causing G240R variant. Homozygosity for the P278KY variant is embryonically lethal. Heterozygous expression of P278KY GlyRS (*Gars1^P278KY/+^*) causes neuromuscular dysfunction in mice by 3 weeks of age, and a high mortality rate by 6–8 weeks. *Gars1^P278KY/+^* mice show progressive neuromuscular junction (NMJ) denervation and length-dependent loss of peripheral sensory and motor axons. Nerve conduction velocity (NCV) is significantly decreased in *Gars1^P278KY/+^* mice, resulting from loss of large-diameter axons rather than myelination defects. Mice that survive past 8 weeks display only moderate disease progression beyond this timepoint. *Gars1* mRNA levels are unaffected by the P278KY mutation, and aminoacylation levels measured from brain tissue of *Gars1^P278KY/+^* mice are comparable to wildtype mice.

While the *Gars1^P278KY/+^* model recapitulates key hallmarks of CMT2D pathology, the phenotype is more severe than in human patients. A second CMT2D mouse model with a milder neuropathy phenotype (*Gars1^C201R/+^*) was developed using N-ethyl-N-nitrosourea (ENU) mutagenesis (Achilli et al., 2009). By 1 month of age, *Gars1^C201R/+^* mice show a reduction in grip strength, hindlimb muscle force, and fine motor control. As with human patients, certain muscles are more affected than others, and the severity of muscle weakness directly corresponds to denervation of the NMJ. The number of motor neurons does not differ between *Gars1^C201R/+^* and wildtype mice, indicating that the NMJ denervation in *Gars1^C201R/+^* mice is the result of axonal degeneration rather than cell death. No demyelination was observed in the Aβ fibers of the saphenous nerve, however, *Gars1^C201R/+^* mice have smaller diameter axons than wildtype mice, resulting in a slower sensory NCV and a reduction in nerve excitability. As with P278KY, homozygosity for the C201R mutation is lethal. While a small number of homozygous *Gars1^C201R^* mice are born, none of these animals survive past postnatal day (P) 17. Unlike *Gars1^P278KY/+^* mice, *Gars1^C201R/+^* mice have a normal lifespan. Analysis of brain tissue homogenates at P15 showed a significant, possibly compensatory, increase in GlyRS protein levels in heterozygous and homozygous *Gars1^C201R^* mice compared to wildtype mice, which normalized by 3 months. There were no significant differences in GlyRS aminoacylation activity between heterozygous and wildtype mice at P15 or P90, but homozygous *Gars1^C201R^* mice displayed a 60% reduction in enzyme activity at P15.

A third mouse model was developed through the introduction of a patient mutation (p.Glu299_Gln302del) into the mouse genome (*Gars1^delETAQ^*) (Morelli et al., 2019). The four amino acid residues that are deleted in the *Gars1^delETAQ^* variant are located within the glycine-binding pocket of GlyRS. GlyRS protein levels are unaffected by the mutation, as measured in both patient and mouse cells. To allow for testing of allele-specific RNAi of mutant *Gars1* as a potential treatment strategy for CMT2D, the *Gars1^delETAQ/+^* mouse strain was crossed with mice homozygously carrying exon 8 of the human *GARS1* gene (*Gars1^huEx8^*), producing compound heterozygous *Gars1^delETAQ/huEx8^* mice. At 12 weeks of age, *Gars1^delETAQ/huEx8^* mice demonstrate reduced grip strength and decreased bodyweight. The mutant mice also display decreased axon diameter, reduced motor and sensory NCV, and variable NMJ denervation in distal muscles, as previously seen in the *Gars1^P278KY/+^* and *Gars1^C201R/+^* mice. Unlike the other two *Gars1* variants, *Gars1^delETAQ^* retains less than 0.01% aminoacylation activity. Adeno-associated virus (AAV)-mediated RNAi of the mutant *Gars1^delETAQ^* allele successfully alleviated the pathological phenotype of *Gars1^delETAQ/huEx8^* mice.

Adenovirus-mediated expression of the patient variants p.Leu129Pro and p.Gly240Arg (*GARS1^L129P^* and *GARS1^G240R^*) in spinal cord and dorsal root ganglion (DRG) neurons has also been used to model CMT2D neuropathy in mice (Lee et al., 2014). The adenovirus was injected into the sciatic nerve of wildtype adult C57BL/6 mice, with expression of FLAG-tagged human *GARS1* variants under control of the mouse choline acetyltransferase (ChAT) promoter, and expression of a GFP reporter under a cytomegalovirus (CMV) promoter. To prevent mitochondrial expression of mutant GlyRS, the mitochondrial targeting sequence was removed from the *GARS1* sequence. Mice expressing *GARS1^L129P^* and *GARS1^G240R^* exhibit allodynia and elevation of multiple markers of peripheral nerve injury and neuropathic pain, including increased microglia, expression of activating transcription factor 3 (ATF3), and activation of p44/42 MAPK (Erk1/2) in mutant *GARS1*-expressing DRG neurons (Lee et al., 2014). The pathogenic *GARS1* mutations also appear to cause a localization defect of GlyRS within peripheral neurons. G240R mutant GlyRS was absent from axons within the sciatic nerve compared to the uniform distribution of wildtype GlyRS throughout the axon, in addition to reduced expression of G240R GlyRS in the central processes of DRG neurons (Lee et al., 2014; Seo et al., 2014b). Likewise, distribution of L129P GlyRS was defective in projections of DRG neurons and peripheral axons, as well as in processes of the dorsal horn of the spinal cord (Seo et al., 2014a). Expression of mutant and wildtype GlyRS was unaltered in motor and sensory neuron cell bodies in the dorsal and ventral horns of the spinal cord (Seo et al., 2014a,b). Consistent with the clinical presentation of CMT2D, no myelination defects were observed in mice overexpressing mutant *GARS1* (Seo et al., 2014a,b).

#### 2.1.2. Genetic modifiers of CMT2D

Interestingly, the severity of the *Gars1^P278KY^* and *Gars1^C201R^* mutations differ between mice from different genetic backgrounds, mirroring the clinical variability among patients. The *Gars1^C201R^* mutation results in a more severe phenotype on the C57BL/6 background than in C3H mice (Achilli et al., 2009), and the average lifespan of *Gars1^P278KY/+^* mice differs between strains (Seburn et al., 2006). These results have methodological implications, highlighting the importance of considering genetic backgrounds when producing mouse models, and when evaluating or comparing results between studies. Additionally, as discussed by Achilli et al. (2009), the heterogeneity between strains implies the presence of genetic modifiers of the disease phenotype, which could be used as potential treatment targets once identified (Achilli et al., 2009). A more recent study identified genes that modified aspects of the abnormal phenotypes elicited in *Drosophila* by both GlyRS and TyrRS variants associated with neuropathy (Ermanoska et al., 2014). Although the specific modifier genes identified in this study do not have vertebrate orthologs, it is probable that there are other genetic modifiers, which account for the broad disease spectrum in human patients. If these modifiers are shared across different ARS1-related neuropathies, they could represent broadly applicable therapeutic targets.

#### 2.1.3. Cellular dysfunction underlying motor and sensory deficits in CMT2D

Animal models have provided functional insights into CMT2D progression at the cellular level. Despite the later onset of CMT2D during adolescence or adulthood, expression of mutant GlyRS impairs early nervous system development in both *Drosophila* and mice. Ubiquitous expression of the *GlyRS^G240R^* variant in *Drosophila* causes high rates of mortality during the late pupal stage, and expression of the severe *GlyRS^P234KY^* variant (equivalent to mouse *Gars1^P278KY^*) is lethal during the first instar (Ermanoska et al., 2014). Conditional expression of *GlyRS^G240R^* in the nervous system of *Drosophila* causes progressive motor deficits, and expression of *GlyRS^P234KY^* within the nervous system is lethal in the final stages of development, further demonstrating the neurotoxicity of CMT2D-associated GlyRS variants (Ermanoska et al., 2014). Interestingly, mesoderm- or muscle-specific expression of *GlyRS^P234KY^* in *Drosophila* perturbs axonal branching in the peripheral nervous system during development (Grice et al., 2018), and leads to an abnormal accumulation of mutant GlyRS at the NMJ pre-synapse (Grice et al., 2015, 2018), indicating that mutant GlyRS also exerts non-cell autonomous toxicity.

Longitudinal characterization of the NMJs of *Gars1^P278KY/+^* and *Gars1^C201R/+^* mice has shown that abnormal development of the NMJ synapse precedes the progressive denervation of distal hindlimb muscles (Sleigh et al., 2014). Among axons of similar length, the severity of denervation correlates with levels of postnatal synaptic growth, suggesting that developmental demands exacerbate the consequences of neuropathy associated GlyRS mutations (Sleigh et al., 2020b). *Gars1^P278KY/+^* mice have a reduced number of mitochondria within NMJ presynaptic terminals, which may account for their inability to meet high energy demands during development (Spaulding et al., 2016). Since GlyRS is a bifunctional ARS, this mitochondrial deficiency could arise directly from impaired mitochondrial GlyRS function.

Overexpression of mutant GlyRS in *Drosophila* either during or post-development impairs synaptic transmission in the giant fiber (GF) circuit (Ermanoska et al., 2014), and both *Gars1^P278KY/+^* and *Gars1^C201R/+^* mice exhibit progressive deficits in NMJ synaptic transmission that correlate with phenotype severity (Spaulding et al., 2016). Presynaptic neurons at the affected NMJs of CMT2D mice have abnormally thin axons and small presynaptic terminals containing fewer vesicles than those of wildtype mice (Spaulding et al., 2016). Likewise, expression of mutant GlyRS in *Drosophila* causes thinning of the GF axon, as well as vacuolization throughout the axon and presynaptic terminal (Ermanoska et al., 2014). Increasing activation of postsynaptic receptors through intraperitoneal administration of physostigmine improves motor performance in CMT2D mice, confirming that defects in synaptic transmission contribute to the pathological phenotype, and identifying a potential treatment target (Spaulding et al., 2016).

In contrast to the ongoing deterioration of motor neurons, developmental abnormalities in sensory neurons of CMT2D mice are evident from birth and appear to be nonprogressive (Sleigh et al., 2020a). Specifically in lumbar DRGs of *Gars1^C201R/+^* and *Gars1^P278KY/+^* mice, there is an abnormally large proportion of nociceptive neurons relative to mechanosensitive and proprioceptive neurons, and nociceptive neurons of the hind paws exhibit impaired arborization (Sleigh et al., 2017a, 2020a). CMT2D mice additionally show increased expression of capsaicin receptors in thermal nociceptor neurons. The severity of these cellular abnormalities correlate with the relative severity of the *Gars1^C201R/+^* and *Gars1^P278KY/+^* mutant phenotypes. The sensory developmental deficits of CMT2D mice manifest behaviorally as hypersensitivity to thermally nociceptive stimuli, and reduced mechanosensation (Sleigh et al., 2017a). Analysis of GlyRS expression levels in DRG neurons of *Gars1^C201R/+^* and *Gars1^P278KY/+^* mice revealed a preferential increase of GlyRS in lumbar mechanosensitive and proprioceptive neurons, which could represent a compensatory upregulation in response to cellular stress in this particular subpopulation of neurons (Sleigh et al., 2020a).

#### 2.1.4. Contribution of loss of canonical GlyRS function to CMT2D

*In vivo* experiments have demonstrated that compromised GlyRS aminoacylation function can result in peripheral nerve defects. GlyRS functions as a homodimer, and homozygous expression of the dimerization-incapable *gars1^T209K^* variant in zebrafish caused a loss of GlyRS aminoacylation function, which led to a decrease in NMJ innervation, impaired muscle development, and abnormal formation of their cardiac valves. A similar phenotype could be produced in wildtype zebrafish through inhibition of protein synthesis between 24 and 96 h post fertilization, suggesting that loss of GlyRS aminoacylation activity impairs protein translation in *gars1^T209K^* zebrafish (Malissovas et al., 2016).

Despite the apparent sensitivity of NMJ development to impaired protein synthesis, the precise relationship between CMT2D pathogenesis and loss of GlyRS canonical function remains unclear, as not all CMT2D-causing *GARS1* variants impair aminoacylation activity (Nangle et al., 2007). *Gars1^P278KY/+^* mice exhibit a severe neuropathy phenotype despite retaining normal aminoacylation function and GlyRS expression levels (Seburn et al., 2006). Likewise, analysis of brain lysates from *Gars1^C201R/+^* mice showed comparable levels of aminoacylation activity to wildtype mice (Achilli et al., 2009). In contrast, despite reducing *Gars1* expression by 50%, heterozygous *Gars1* knockout in mice (*Gars1^XM256/+^*) does not trigger a disease phenotype (Seburn et al., 2006), demonstrating that the neuropathy is not caused by haploinsufficiency.

However, despite retaining normal aminoacylation capacity *in trans* to wildtype GlyRS, neither the *Gars1^P278KY^* nor the *Gars1^C201R^* variants can complement a loss-of-function allele (Achilli et al., 2009; Motley et al., 2011). The canonical role of GlyRS in protein translation could potentially be disrupted through alternative mechanisms, such as mislocalisation or decreased accuracy in tRNA charging. Multiple CMT2D GlyRS variants impair the transport of GlyRS into neurite projections *in vitro* (Antonellis et al., 2006; Nangle et al., 2007). The *in vivo* effects of CMT2D mutations on GlyRS localization are inconclusive (Stum et al., 2011; Lee et al., 2014; Seo et al., 2014a,b; Niehues et al., 2015), and it remains to be seen if mislocalisation of mutant GlyRS contributes to the CMT2D phenotype. Mischarging of tRNA by mutant GlyRS could conceivably also lead to neurodegeneration through toxic accumulation of misfolded proteins, which is a major pathological hallmark of many neurodegenerative diseases including amyotrophic lateral sclerosis (Parakh and Atkin, 2016). However, there is so far no evidence of misfolded proteins aggregating *in vitro* (Antonellis et al., 2006) or within the nervous system of CMT2D mice (Stum et al., 2011) arguing against tRNA mischarging as the primary cause of CMT2D pathophysiology.

Since GlyRS is a bifunctional ARS, mitochondrial dysfunction might contribute to the CMT2D pathophysiology. Abnormal mitochondrial dynamics and impaired mitochondrial trafficking have previously been implicated in the pathogenesis of multiple axonal neuropathies (McCray and Scherer, 2021). Whilst several mitochondrial abnormalities were observed within induced neuronal progenitor cells (iNPCs) generated from fibroblasts of a DSMAV patient, these were not detected in *Gars1^C201R/+^* mice (Boczonadi et al., 2018). Additionally, expression of mutant GlyRS exclusively in the cytosol is sufficient to trigger peripheral neuropathy in *Drosophila* (Ermanoska et al., 2014) and mice (Lee et al., 2014; Seo et al., 2014a,b), suggesting that mitochondrial dysfunction is not the primary cause of CMT2D neuropathy. Nevertheless, mitochondrial deficits have been observed in *Gars1^P278KY/+^* mice, including a reduction in the number of mitochondria at synaptic terminals of hindlimb NMJs (Spaulding et al., 2016), and decreased oxidative phosphorylation (OXPHOS) in the gastrocnemius muscle (Ozes et al., 2021). Together, these results suggest that while neuropathy can occur independently of mitochondrial dysfunction, this may still be a contributing factor to the CMT2D pathology.

Although many of the GlyRS variants identified to date exhibit loss-of-function characteristics (Griffin et al., 2014; Zhang et al., 2021b), a common loss-of-function mechanism has not yet been identified among the CMT2D GlyRS variants. This divergence could account for some of the heterogeneity in clinical presentations among patients carrying different GlyRS mutations.

#### 2.1.5. Toxic GlyRS gain-of-function

Irrespective of their effects on normal GlyRS function, there is converging evidence that pathogenic GlyRS mutations exert toxic gain-of-function effects. Overexpression of mutant GlyRS on the background of active endogenous GlyRS in *Drosophila* induces a CMT2D phenotype (Ermanoska et al., 2014), and expression of the *Gars1^C201R^* and *Gars1^P278KY^* variants in mice show dose-dependent toxicity, which cannot be counteracted with overexpression of wildtype GlyRS (Motley et al., 2011). In addition, a subsequent study demonstrated that the neuropathy phenotypes of both *Gars1^P278KY/+^* and *Gars1^delETAQ/+^* mice could be rescued through AAV-mediated RNAi of the mutant alleles, supporting the toxic gain-of-function hypothesis, and providing proof-of-concept for an AAV-mediated gene therapy approach for CMT2D (Morelli et al., 2019).

Many of the disease associated *GARS1* mutations cause conformational changes, exposing new surfaces of the GlyRS protein, which enable abnormal interactions with other proteins (He et al., 2011, 2015). Studies conducted in both mice and *Drosophila* have provided evidence of aberrant interactions between mutant GlyRS and multiple novel binding partners contributing to the neuropathy phenotype. These results are discussed in the following sections.

##### 2.1.5.1. Aberrant binding of Nrp1 and Trk receptors

Consistent with the developmental defects observed in CMT2D animal models, pathogenic GlyRS variants interfere with signaling pathways that are important for nervous system development. Unlike wildtype GlyRS, several CMT2D causing GlyRS variants strongly bind Neuropilin 1 (Nrp1) *in vitro* (He et al., 2015; Sleigh et al., 2017b; Mo et al., 2018), thereby competing with the binding of vascular endothelial growth factor A (VEGF-A). In mice, the strength of the GlyRS-Nrp1 binding directly correlates with disease severity, with lower affinity between GlyRS and Nrp1 resulting in milder disease (Sleigh et al., 2017b). VEGF-Nrp1 signaling plays a role in vascularization, as well as having a neuroprotective effect in peripheral motor neurons (Oosthuyse et al., 2001; Kofler and Simons, 2015). Treating *Gars1^P278KY/+^* mice with VEGF-A injections into the hindlimbs prior to the onset of neuropathy reduced the severity of motor deficits (He et al., 2015). Future studies should validate whether the pathological effects of other CMT2D variants that bind Nrp1 can similarly be counteracted through VEGF-A administration.

In addition to VEGF-A signaling, Nrp1 also plays a role in semaphorin signaling (Gu et al., 2003). Semaphorins are a family of signaling proteins that control developmental processes of the nervous system including neurogenesis, axonal pathfinding, and synapse formation through interactions with their receptors, which include plexins and neuropilins (Limoni and Niquille, 2021). In *Drosophila* there is no ortholog of the vertebrate Nrp1 gene, with semaphorin signaling occurring via interactions with plexins that are encoded by two genes, *plexA* and *plexB* (Grice et al., 2018). Heterozygous knockout of these genes exacerbates the axonal branching defects caused by overexpression of *GlyRS^P234KY^,* suggesting that disrupted plexin signaling may underlie the developmental abnormalities seen in *GlyRS^P234KY^ Drosophila* (Grice et al., 2018). Additionally, interactions between mutant GlyRS and plexB contribute to the accumulation of GlyRS at the NMJ presynaptic terminal and interfere with the capacity of plexB to bind semaphorin-2a. This interaction directly contributes to the neuropathy phenotype, as overexpression of semaphorin-2a attenuates the pathology in *GlyRS^P234KY^ Drosophila* (Grice et al., 2018). Restoration of semaphorin signaling could therefore also be an avenue for treatment of CMT2D, which should be further explored in mammalian disease models.

The abnormal development of sensory neurons in CMT2D mice is hypothesized to be the result of aberrant activation of tropomyosin receptor kinase (Trk) receptors by mutant GlyRS (Sleigh et al., 2017a). The binding of neurotrophins to Trk receptors regulates key processes including neuronal growth, migration and survival (Huang and Reichardt, 2003), and has directly been implicated in the differentiation of sensory neuron subtypes during development (Moqrich et al., 2004). *In vitro* results demonstrate that mutant GlyRS binds to and activates Trk receptors A-C, and that the extent of variant binding correlates with the severity of the CMT2D phenotype observed in mice (Sleigh et al., 2017a). The functional consequences of GlyRS-Trk binding are yet to be tested *in vivo*, however, it is plausible that this interaction disrupts sensory neuron development in CMT2D (Sleigh et al., 2017a, 2020a).

##### 2.1.5.2. Axonal transport deficits due to excessive *α*-tubulin deacetylation

*In vivo* studies have demonstrated that impaired axonal transport contributes to CMT2D pathology. Axonal transport deficits are a common feature of several neurodevelopmental and neurodegenerative diseases including CMT neuropathies, owing to the reliance of neurons on efficient and reliable shuttling of various cargoes between the soma and synapses to support cellular function and viability (Sleigh et al., 2019). Excessive deacetylation of *α*-tubulin has been demonstrated in multiple CMT2D models, disrupting the microtubule cytoskeleton, which forms the basis of axonal transport. Several CMT2D GlyRS mutants aberrantly bind histone deacetylase 6 (HDAC6) and enhance its activity, significantly decreasing *α*-tubulin acetylation in peripheral nerves (Benoy et al., 2018; Mo et al., 2018). Another target of HDAC6 is the mitochondrial Rho GTPase 1 (MIRO1), a protein which helps to facilitate mitochondrial transport along the axons and dendrites of motor and sensory neurons (Nguyen et al., 2014; Kalinski et al., 2019). HDAC6 activity has previously been linked to neuropathy through excessive deacetylation of MIRO1 (English and Barton, 2021), and this pathway may similarly be involved in the pathogenesis of CMT2D.

DRG neurons from *Gars1^P278KY/+^* mice exhibit impairments in axonal transport prior to the onset of neuromuscular dysfunction, implying that these transport defects are not secondary consequences of neurodegeneration (Mo et al., 2018). An assay of axonal transport dynamics in DRGs of late-symptomatic *Gars^C201R/+^* mice showed impaired anterograde and retrograde mitochondrial transport (Benoy et al., 2018), possibly accounting for the reduction in mitochondria observed at NMJs of CMT2D mice (Spaulding et al., 2016). HDAC6 inhibition in symptomatic CMT2D mice restored axonal transport in DRG neurons and improved both sensory and motor function (Benoy et al., 2018; Mo et al., 2018). Consistently, HDAC6 inhibition also reversed axonal transport deficits in *GARS1^P234KY^* and *GARS1^P724H^* human induced pluripotent stem cell (iPSC) lines (Smith et al., 2021).

Also contributing to the hypoacetylation of *α*-tubulin in CMT2D mice is impaired inhibition of the NAD-dependent deacetylase sirtuin 2 (SIRT2) by mutant GlyRS. Wildtype GlyRS binds SIRT2, thereby inhibiting its deacetylation activity. CMT2D GlyRS variants have reduced binding affinity for SIRT2, leading to an increase in SIRT2 deacetylase activity (Zhao et al., 2021). Similar to results obtained with HDAC6 inhibition, SIRT2 inhibition improved motor function in *GlyRS^G526R^ Drosophila*, and extended their lifespan (Zhao et al., 2021). Together, these results provide compelling evidence that axonal transport deficits arising from increased *α*-tubulin deacetylation contribute to the pathophysiology of certain forms of CMT2D, and that the deacetylases HDAC6 and SIRT2 may be actionable therapeutic targets.

An important task for future studies will be to determine the extent to which axonal transport defects generalize across CMT-associated GlyRS variants, and more broadly to other neuropathies caused by ARS1 mutations. In contrast to previous studies, a recent study failed to detect the defects in retrograde axonal transport of signaling endosomes in the DRGs of early symptomatic *Gars1^C201R/+^* mice (Sleigh et al., 2020b). Numerous possible explanations for this discrepancy were discussed by the authors, including measurement of different cellular cargoes between studies, and analysis of cells cultured from mice at different stages of the disease. Nevertheless, this apparent inconsistency warrants further investigation, and the use of intravital imaging techniques would circumvent some of the inherent shortcomings of *in vitro* and *ex vivo* assays (Sleigh et al., 2017c).

#### 2.1.6. Impaired protein synthesis caused by sequestration of tRNA^Gly^

The established CMT2D animal models have also provided evidence that CMT2D-associated GlyRS mutations impair protein synthesis through mechanisms indirectly related to defective tRNA charging. Neurons are particularly vulnerable to disrupted protein synthesis, with inhibition of protein synthesis being sufficient to induce a neuropathy phenotype (Chihara et al., 2007; Malissovas et al., 2016). Ubiquitous overexpression of the G240R and G526R GlyRS variants in adult *Drosophila* reduced the rate of protein synthesis in both motor and sensory neurons (Niehues et al., 2015), and translation was similarly impaired in motor neurons of *Gars1^P278KY/+^* and *Gars1^C201R/+^* mice (Spaulding et al., 2021). This impairment in protein synthesis is caused by sequestration of tRNA^Gly^ by mutant GlyRS, with overexpression of tRNA^Gly^ rescuing peripheral neuropathy in multiple *Drosophila* and mouse CMT2D models (Zuko et al., 2021).

The sequestration of tRNA^Gly^ directly impacts the elongation stage of protein synthesis and has a secondary effect on the initiation of translation, through activation of the integrated stress response (ISR) (Mendonsa et al., 2021). The absence of tRNA^Gly^ at the ribosome during translation causes stalling of the ribosome at glycine codons. Within alpha motor neurons of the spinal cord, as well as in a subset of sensory neurons, pausing of the ribosome induces phosphorylation of the alpha subunit of eukaryotic initiation factor 2 (eIF2a) by the sensor kinase general control nondepressible 2 (GCN2), triggering the ISR (Mendonsa et al., 2021; Spaulding et al., 2021). Genetic deletion or pharmacological inhibition of GCN2 prevents ISR activation and improves both sensory and motor function in CMT2D mice (Spaulding et al., 2021). The precise link between induction of the ISR and neuropathy remains unclear, as does the specificity of the cell populations affected, however, the phenotypic improvement in CMT2D mice following GCN2 inhibition suggests that this may be an effective treatment target (Spaulding et al., 2021).

#### 2.1.7. Neurotrophin 3 gene therapy for CMT2D

Neurotrophin 3 (NT-3) gene therapy has been employed to improve the phenotype of CMT2D mice (Ozes et al., 2021). NT-3, through TrkC signaling, supports several aspects of nervous system development and function, including Schwann cell migration, peripheral neurite outgrowth, and development of the NMJ (Yamauchi et al., 2004; Sheard et al., 2010). Intramuscular delivery of NT-3 has previously been shown to alleviate neuropathy in mouse models of CMT type I (Sahenk et al., 2014). AAV1-mediated delivery of NT-3 (AAV1.NT-3) into the gastrocnemius muscles of post-symptomatic *Gars1^P278KY/+^* mice led to improvements in motor performance, increased CMAP, increased myelin thickness, and improved NCV (Ozes et al., 2021). Additionally, NMJ denervation was reduced and mitochondrial abnormalities in the muscles of *Gars1^P278KY/+^* mice were corrected following AAV1.NT-3 treatment. Improvements were less pronounced in *Gars1^delETAQ/huEx8^* mice, possibly due to their milder phenotype, or due to treatment at a later timepoint (Ozes et al., 2021).

### 2.2. Dominant *YARS1* mutations and disease

Mutations in the cytosolic tyrosyl-tRNA synthetase (TyrRS) gene *YARS1* are associated with dominant-intermediate CMT type C (DI-CMTC). The age of onset for DI-CMTC is highly variable, with reports ranging between 5 and 60 years of age (Jordanova et al., 2006; Hyun et al., 2014; Thomas et al., 2016). Patients typically present with slowly progressive, mild-to-moderate motor and sensory deficits, including distal muscle weakness and atrophy, as well as impaired sensation of vibration and pinprick stimuli. The motor symptoms are generally more pronounced than the sensory impairments. Most DI-CMTC patients have abnormal MNCVs, with reported median MNCVs widely ranging between 25 and 58 m/s. The underlying cause of MNCV slowing in DI-CMTC patients is yet to be determined, with some evidence of both axonal loss and demyelination observed in patient nerve biopsies (Thomas et al., 2016).

#### 2.2.1. Effects of pathogenic *YARS1* mutations

##### 2.2.1.1. Canonical enzyme function

As was the case for GlyRS, 50% loss of TyrRS activity does not produce a neuropathy phenotype in either *Drosophila* or mice (Storkebaum et al., 2009; Hines et al., 2021). *YARS1* variants have varied impacts on the efficiency of the aminoacylation reaction itself. For example, whilst the *YARS1^G41R^* patient variant reduces the tRNA^Tyr^ aminoacylation rate by 34% (Jordanova et al., 2006), the *YARS1^E196K^* patient variant has a comparable rate of aminoacylation to the wildtype enzyme, despite causing a severe motor phenotype when overexpressed in *Drosophila* (Storkebaum et al., 2009; Froelich and First, 2011). Both variants are capable of dimerizing with wildtype TyrRS, and results from a yeast complementation assay suggest that the TyrRS mutants may exert a dominant-negative effect on the wildtype enzyme function (Jordanova et al., 2006).

##### 2.2.1.2. Localization

GlyRS and TyrRS both show distinctive subcellular localization patterns within peripheral axons and Schwann cells, possibly reflecting localized demands for protein synthesis, or secondary enzyme functions specific to these locations (Jordanova et al., 2006; Stum et al., 2011). *In vitro*, wildtype TyrRS is located in granular structures concentrated within the outgrowing axon terminals of differentiating mouse N2a and human SH-SY5Y neuroblastoma cell lines, whereas mutant (p.Gly41Arg and p.Glu196Lys) TyrRS shows a homogenous, non-granular distribution pattern (Jordanova et al., 2006). Despite this *in vitro* result, mislocalisation of mutant TyrRS was not observed in a *Drosophila* model of DI-CMTC (Niehues et al., 2015).

##### 2.2.1.3. Novel protein interactions of mutant TyrRS

Like CMT2D-associated GlyRS mutations, DI-CMTC mutations can induce conformational changes in TyrRS, which expose previously hidden surfaces of the enzyme, enabling novel interactions with other proteins to take place (Blocquel et al., 2017). DI-CMTC linked *YARS1* variants (*YARS1^G41R^*, *YARS1^E196K^*, and *YARS1^del153–156^*) sequester tripartite motif-containing 28 (TRIM28), leading to hyperactivation of the transcription factor E2F1 in mammalian cells (Blocquel et al., 2017; Bervoets et al., 2019). Transcriptomic analyses of a DI-CMTC *Drosophila* model also suggest that abnormal interactions occur between mutant TyrRS and multiple transcription factors within the nucleus (Bervoets et al., 2019). The transcriptional dysregulation caused by these interactions have been shown to contribute to the DI-CMTC phenotype in *Drosophila*.

#### 2.2.2. Animal models of DI-CMTC

The *Drosophila* homolog of *YARS1* (*TyrRS*) is 68% identical to the human gene, and wildtype human TyrRS can compensate for loss of *Drosophila* TyrRS, indicating functional conservation between species (Storkebaum et al., 2009). Ubiquitous expression of a non-pathogenic TyrRS variant (p.Lys256Asp) in *Drosophila* does not produce an abnormal phenotype, whereas expression of neuropathy-linked TyrRS variants in *Drosophila* cause dose-dependent toxicity, with strong ubiquitous expression of DI-CMTC variants leading to developmental lethality (Storkebaum et al., 2009; Leitão-Gonçalves et al., 2012).

Selective expression of pathogenic TyrRS variants in neurons is sufficient to cause a DI-CMTC phenotype including progressive motor dysfunction with no signs of degeneration in the brain or ventral nerve cord (Storkebaum et al., 2009). Interestingly, in contrast to the *Drosophila* models of CMT2D discussed previously, conditional expression of DI-CMTC variants in muscle does not produce a disease phenotype, suggesting that the motor dysfunction in the DI-CMTC model originates in neuronal cells. Expression of DI-CMTC variants within the GF circuit impairs synaptic transmission between the GF and the tergotrochanteral motoneuron (TTMn), and causes thinning and progressive degeneration of the GF axon terminal (Storkebaum et al., 2009). Selective presynaptic, but not postsynaptic expression of the TyrRS variants reproduced these defects. DI-CMTC *Drosophila* also show distinct morphological abnormalities in their eyes, wings, and bristles, which are unrelated to the clinical phenotype but are nonetheless useful markers of cellular dysfunction (Storkebaum et al., 2009).

A mammalian model of DI-CMTC has been generated through expression of the patient variant p.Glu196Lys in mice (*Yars1^E196K^*) (Hines et al., 2021; Spaulding et al., 2021). Homozygous *Yars1^E196K/E196K^* mice exhibit a pronounced neuropathy phenotype, with increasingly impaired performance in the wire hang test from 2 months of age and significantly reduced sciatic NCV from 4 months of age. In contrast, heterozygous *Yars1^E196K/+^* mice do not develop an overt neuropathy phenotype, indicating that expression of this variant is better tolerated in mice than in humans. Interestingly, a similar species difference in phenotype severity has also been observed in knock-in mouse models for recessive ARS disease (Fröhlich et al., 2020; Klugmann et al., 2022). No defects in myelination were observed in homozygous or heterozygous *Yars1^E196K^* mice, however, *Yars1^E196K/E196K^* mice showed a non-progressive decrease in peripheral motor axon diameter compared to wildtype mice at 4 months of age (Hines et al., 2021). Expression of the *Yars1^E196K^* allele in combination with a *Yars1* null allele (*Yars1^E196K/−^*) produced a similar phenotype to *Yars1^E196K/E196K^*, with an additional characteristic of weight loss.

#### 2.2.3. Dysregulation of transcription and translation in DI-CMTC

DI-CMTC-associated TyrRS variants can cause transcriptional dysregulation through novel interactions with transcription factors in the nucleus. In both DI-CMTC patient cells and in *Drosophila*, the transcription factor E2F1 is hyperactivated in the presence of mutant TyrRS (Bervoets et al., 2019). Inhibition of E2F1 improved developmental survival rates of *Drosophila* expressing *YARS1^E196K^* but was not sufficient to rescue the motor defects. Excluding TyrRS from the nucleus prevented the DI-CMT phenotype in *YARS1^E196K^* expressing *Drosophila* and normalized their gene expression profile, suggesting that aberrant interactions between mutant TyrRS and transcription factors within the nucleus are likely triggering the DI-CMTC pathophysiology (Bervoets et al., 2019). Given that other cytosolic ARSs have also been found within the nucleus, it will be of interest to determine whether transcription is similarly dysregulated when these are mutated.

Furthermore, reduced rates of protein synthesis have been observed in *Drosophila* DI-CMTC models, mirroring results obtained from *Drosophila* models of CMT2D (Niehues et al., 2015). Additionally, as for CMT2D mice, activation of the ISR was detected in homozygous *Yars1^E196K^* mice, however the therapeutic benefit of GCN2 inhibition is yet to be tested in this model (Spaulding et al., 2021).

### 2.3. Dominant *HARS1* mutations and disease

Dominant mutations in *HARS1,* the gene encoding the cytosolic histidyl-tRNA synthetase (HisRS), have predominantly been associated with Charcot-Marie-Tooth disease type 2W (CMT2W) and hereditary motor neuropathy (HMN), with both disease presentations sometimes occurring within the same family (Safka Brozkova et al., 2015). As with mutations in the other ARS1 genes, disease severity is highly variable between individuals. Additionally, there has been one report of a patient presenting with peripheral neuropathy, accompanied by periodic acid-Schiff (PAS) positive intra-axonal inclusions and cerebellar atrophy (Royer-Bertrand et al., 2019). However, since this is so far an isolated case, it is not clear whether these symptoms were caused by an additional genetic factor or whether they occurred as a direct consequence of the *HARS1* mutation (Royer-Bertrand et al., 2019).

#### 2.3.1. Effects of pathogenic *HARS1* mutations

All disease-causing dominant *HARS1* mutations are located within the catalytic domain of HisRS and have each shown loss-of-function characteristics *in vitro*. For several pathogenic HisRS variants, the reduced rate of aminoacylation is attributable to an elevated Michaelis constant (K_M_) for binding ATP and histidine, indicating impairment of the mutant enzymes to recognize these substrates (Abbott et al., 2018). Despite the apparent loss of canonical enzyme function, neither the inability of mutant HisRS to support yeast growth in a yeast complementation assay, nor the decrease of aminoacylation activity within patient cells correlate with disease severity (Safka Brozkova et al., 2015; Blocquel et al., 2019). Localization of CMT2W-associated *HARS1* mutants is normal within differentiated rat pheochromocytoma (PC12) cells and *C. elegans* motor neurons, respectively (Vester et al., 2013; Mullen et al., 2021). Dimerization of the mutant enzymes also appears unaffected (Abbott et al., 2018; Mullen et al., 2021). Consistent with the tissue-specific disease phenotype, HisRS mutants have cell type-specific impacts on protein synthesis *in vitro*. While protein synthesis was unchanged in fibroblasts from a CMT2W patient, expression of CMT2W *HARS1* mutants in PC12 cells impaired protein synthesis and induced the ISR, causing impaired neurite outgrowth (Royer-Bertrand et al., 2019; Mullen et al., 2021).

#### 2.3.2. Animal models of CMT2W

Studies conducted *in vivo* have further confirmed the neurotoxicity of CMT-causing HisRS variants and highlighted the sensitivity of the nervous system to deficiencies in protein synthesis that may arise from these mutations. In *C. elegans*, overexpression of CMT2W *hars-1* variants in GABAergic motor neurons is neurotoxic, causing progressive morphological defects in axons and concomitant locomotor deficits (Vester et al., 2013; Safka Brozkova et al., 2015). Similarly, ubiquitous overexpression of CMT HisRS variants in zebrafish disrupts axonal morphology, causing shortening and disorganization of DRG projections as well as an abnormal motor phenotype (Mullen et al., 2021). So far, no mammalian models of CMT2W have been described.

#### 2.3.3. Elucidating the dominant *HARS1* disease mechanism

Heterozygous *Hars1* knockout mice have not been characterized, so HisRS haploinsufficiency cannot yet be ruled out as a cause of CMT2W. Another possibility is that CMT2W-causing HisRS variants exert a dominant-negative effect on the activity of wildtype HisRS, thus lowering tRNA^His^ aminoacylation below a critical threshold that is required to support normal development and function of peripheral neurons. A dominant-negative effect of mutant HisRS could also possibly compromise non-canonical HisRS functions relevant to supporting peripheral neuron development and function. These hypotheses could be assessed in future *in vivo* studies by investigating whether the pathological phenotype can be rescued through overexpression of wildtype HisRS, or by disrupting the formation of mutant-wildtype HisRS heterodimers (Meyer-Schuman and Antonellis, 2021).

It is also possible that *HARS1* mutations lead to a HisRS conformation change, which could result in aberrant protein interactions, as has been observed with other CMT-ARS1 mutations. Interestingly, an analysis of multiple CMT-associated *HARS1* variants revealed that each mutant protein had some degree of conformational opening compared to wildtype HisRS, and that the degree of structural opening correlated with disease severity (Bervoets et al., 2019), however, no specific abnormal protein interactions for mutant HisRS have yet been identified.

### 2.4. Dominant *AARS1* mutations and disease

Dominant mutations in the cytosolic Alanyl-tRNA synthetase (AlaRS) gene *AARS1* primarily lead to an axonal form of CMT (CMT2N). Neuropathies associated with *AARS1* mutations have a highly variable age of onset and clinical severity, with some patients exhibiting reduced NCV diagnosed as DI-CMT. One dominant *AARS1* variant has also been linked to dHMN, distinguished from CMT2N by the lack of sensory involvement (Zhao et al., 2012). Another *AARS1* variant was found to cause a mild myelopathy in addition to axonal neuropathy (Motley et al., 2015), further expanding the disease spectrum associated with dominant *AARS1* mutations.

A dominant mutation in *AARS1* has also been linked to the leukoencephalopathy HDLS-S (Sundal et al., 2019). HDLS-S presents in adulthood and has a rapid and severe degenerative course, with a median life expectancy of less than 10 years following disease onset. Unlike CMT2N, HDLS-S patients do not show any signs of peripheral axonal degeneration, suggesting that the underlying pathomechanism is different. The responsible *AARS1* mutation is located within the aminoacylation domain, and while AlaRS expression levels are unaffected, protein function has not yet been assessed for this variant.

Animal models for neuropathy causing AlaRS variants are sparse, with only a single study in zebrafish conducted to date (Weterman et al., 2018). Several *in vitro* studies have provided intriguing results that should be followed up *in vivo*. These key findings are briefly discussed in the following sections.

#### 2.4.1. Effects on canonical enzyme function

The AlaRS protein structure comprises an aminoacylation domain, an editing domain, and the C-terminal domain (C-Ala). AlaRS functions as a monomer in human cells, however, the protein is capable of dimerization, possibly to facilitate non-canonical activities (Sun et al., 2016). In humans, the C-Ala domain is not required for aminoacylation but has been shown to bind DNA within the nucleus and to mediate AlaRS dimerization, suggesting that it is involved in the functional expansion of this enzyme (Sun et al., 2016; Zhang et al., 2021a).

Neuropathy-causing *AARS1* mutations have been identified in each of the AlaRS domains. Consistent with other ARS1 variants linked to CMT disease, the occurrence of neuropathy appears to be uncoupled from loss of aminoacylation function (Sun et al., 2021). This was further demonstrated in a zebrafish study, where the expression of either hypomorphic or hypermorphic CMT2N-causing *AARS1* variants in 1-cell stage embryos caused severely abnormal embryonic development and aberrant peripheral nerve patterns (Weterman et al., 2018). The *AARS1* mutation most commonly linked to CMT (p.Arg329His; *AARS1*^R329H^) is located within the AlaRS aminoacylation domain but does not impair its canonical function (Sun et al., 2021). While localization of mutant *AARS1*^E778A^ appears normal within MN-1 cells (McLaughlin et al., 2012), the *AARS1*^R329H^ variant exhibits abnormal, punctate co-localization with Golgi bodies and lysosomes in COS-7 fibroblasts, and has an inhibitory effect on neuronal cell differentiation and process outgrowth in N1E-15 cells (Imaizumi et al., 2019). Another AlaRS variant with a mutation in the aminoacylation domain (p.Asn71Tyr) caused similar localization defects and inhibition of neurite outgrowth *in vitro*, which could be prevented through pre-treatment with the antiepileptic drug valproic acid (Tatsumi et al., 2019). Whether these variants cause localization defects *in vivo* is yet to be investigated.

*AARS1* variants that cause neuropathy do not affect proofreading *in vitro*, including mutations located within the AlaRS editing domain (Sun et al., 2021). Furthermore, mice homozygous for editing-defective AlaRS alleles exhibit ataxia due to loss of cerebellar Purkinje neurons, but do not develop a neuropathy phenotype (Lee et al., 2006; Stum et al., 2011), suggesting that tRNA mischarging is not the underlying cause of peripheral neuropathies associated with dominant ARS mutations.

#### 2.4.2. Toxic *AARS1* gain-of-function mutations

Intriguingly, one *in vitro* study found that all mutations located within the AlaRS aminoacylation domain induced a conformational opening in the protein, which led to an aberrant interaction with Nrp1 (Sun et al., 2021), mirroring the interactions previously identified between mutant GlyRS and Nrp1 (He et al., 2015; Mo et al., 2018). However, in contrast to GlyRS and TyrRS mutants, these AlaRS variants did not interact with HDAC6 or TRIM28. An editing domain mutation investigated in the same study (p.Glu688Gly) induced a different conformational opening in AlaRS, which did not enable binding of Nrp1 but might lead to novel, toxic interactions with other proteins. Hence, despite the shared interactions between Nrp1 and specific AlaRS and GlyRS variants, there is a clear heterogeneity in the downstream consequences of neuropathy associated ARS1 mutations, even among mutations in the same gene. Future studies using animal models are required to establish whether the Nrp1-AlaRS interaction occurs *in vivo* and how this interaction contributes to the neuropathy phenotype.

### 2.5. Dominant *WARS1* mutations and disease

Mutations in the *WARS1* gene, which encodes the tryptophanyl-tRNA synthetase (TrpRS), have been linked to autosomal dominant dHMN (Tsai et al., 2017; Wang et al., 2019). Typically, symptoms emerge during adolescence or early adulthood, primarily affecting the distal lower limb muscles, sometimes followed by upper limb involvement. *WARS1* variants may also cause CMT neuropathy, as a number of patients harboring the p.Asp341Gly mutation have been reported to have mild sensory deficits (Wang et al., 2019), however, in the small number of cases identified so far, the pathology seems most severe in motor axons.

#### 2.5.1. Pathogenic *WARS1* mutations *in vitro*

There are currently no published studies on the effects of pathogenic *WARS1* mutations *in vivo*. Both dHMN-linked *WARS1* mutations described in the literature (p.His257Arg and p.Asp341Gly) occur in highly conserved residues of the TrpRS protein. The p.His257Arg mutation exerts a dominant-negative effect on TrpRS aminoacylation activity and on overall protein synthesis *in vitro* (Tsai et al., 2017). Expression of *WARS1^H257R^* in both mouse and human neuronal cell lines impaired neurite outgrowth and caused neurite degeneration (Tsai et al., 2017), consistent with effects observed with other pathogenic ARS1 mutations.

Similar to what has been discovered for GlyRS and TyrRS, pathogenic mutations in TrpRS appear to modulate its interactions with other proteins. TrpRS is known to be involved in angiostasis, and the p.His257Arg variant increases the angiostatic activity of the protein by enhancing the interaction between TrpRS and VE-cadherin (Tsai et al., 2017). *In vivo* experiments may help to determine whether the TrpRS-VE-cadherin interaction contributes to the dHMN phenotype, and hence whether this represents a potential treatment target. It is also possible that neuropathy associated *WARS1* variants cause additional aberrant protein interactions, which may disrupt other cellular processes.

### 2.6. Dominant *MARS1* mutations and disease

Mutations in *MARS1*, the cytosolic methionyl-tRNA synthetase (MetRS) gene are associated with CMT type 2U (CMT2U) (Gonzalez et al., 2013; Hyun et al., 2014; Hirano et al., 2016; Nam et al., 2016; Sagi-Dain et al., 2018; Gillespie et al., 2019). CMT2U patients present with progressive motor deficits that are most pronounced in the lower limbs, often accompanied by decreased sensation in the lower extremities. NCV results are typically consistent with axonal neuropathy, however, peripheral nerve demyelination has also been observed in some individuals (Hirano et al., 2016; Sagi-Dain et al., 2018). The age of onset for CMT2U varies significantly, ranging from early childhood to late adulthood. Disease severity is similarly heterogeneous, even among family members expressing the same variant (Sagi-Dain et al., 2018). Interestingly, the first *MARS1* variant associated with CMT2U (p.Arg618Cys) appeared to have incomplete penetrance (Gonzalez et al., 2013; Rips et al., 2018). However, given the very late disease onset of some patients, it is possible that family members unaffected at the time of testing developed disease later in life.

The *MARS1* variants that have so far been linked to neuropathy occur in highly conserved residues of the protein. *In vitro* and *in vivo* characterization of *MARS1* variants are limited, with no animal models for CMT2U currently available. *MARS1* variants that have been evaluated in yeast complementation assays have shown reduced capacity to support yeast growth, consistent with loss of enzyme function (Gonzalez et al., 2013; Rips et al., 2018; Gillespie et al., 2019). *In silico* modeling of the p.Arg618Cys variant suggested that this mutation induces a structural opening in the protein (Gonzalez et al., 2013), possibly enabling novel protein interactions to take place, as has been observed for other neuropathy-causing ARS1 variants.

### 2.7. Dominant *SARS1* mutations and disease

Recently, 16 patients with CMT from three unrelated families were identified with heterozygous mutations in *SARS1*, the gene encoding cytosolic seryl-tRNA synthetase (He et al., 2023). Patients exhibited progressive muscle weakness and atrophy in their limbs, sensory loss, and NCVs consistent with an intermediate form of CMT. The two pathogenic mutations identified in this cohort occur in highly conserved residues within the aminoacylation domain of SerRS, and result in significantly reduced aminoacylation activity *in vitro.* Both mutant proteins showed increased dimerization with wildtype SerRS. Interestingly, eIF2a phosphorylation was elevated in fibroblasts and N2a cells co-transfected with mutant and wildtype SerRS, but not in cells transfected with mutant SerRS alone (He et al., 2023), suggesting that the formation of mutant-wildtype SerRS heterodimers is pathogenic. Once *in vivo* models of SerRS-linked CMT are established, it will be of interest to determine whether disrupting the dimerization capability of mutant SerRS provides any phenotypic improvement.

## 3. Conclusion

Many *in vivo* models for ARS1-associated neuropathies have been generated through knock-in or overexpression of pathogenic ARS1 alleles in multiple species including *Drosophila*, zebrafish, and mice. Where tested, heterozygous knockout of ARS1 genes in mice has not produced a neuropathy phenotype, implying that these disorders are not caused by haploinsufficiency. The majority of animal models for dominant ARS1 diseases have focused on GlyRS and TyrRS variants, however, results from these studies provide a valuable starting point for investigating the pathophysiology underlying neuropathies caused by mutations in the other ARS1 genes. It is important to note that introducing neuropathy associated ARS1 mutations into a model organism might not always accurately capture the human disease spectrum due to interspecies differences including variations in ARS protein structure, splicing, and signaling pathways. Therefore, supplementing data obtained from animal studies with alternative approaches such as patient-derived iPSC disease models will increase their translational relevance.

Existing *in vivo* models have linked the expression of pathogenic ARS1 variants to defects in the early development of the peripheral nervous system, synaptic transmission deficits at the NMJ, and progressive NMJ denervation. Novel toxic protein interactions have been uncovered for a variety of neuropathy associated ARS1 variants, which directly contribute to the disease pathogenesis, and therefore represent actionable therapeutic targets. *In vivo* studies have additionally demonstrated the vulnerability of peripheral nervous system development and function to disturbances in protein synthesis, which can arise from compromised ARS function. Individual ARS1 variants differ greatly in the extent of their loss-of-function, as well as in their interactions with other proteins. Different cellular pathways are perturbed across ARS1 variants, likely contributing to the great heterogeneity amongst patients and necessitating the development of personalized medicine and tailored treatment strategies. It will therefore be imperative for future studies to characterize the downstream consequences of other pathogenic ARS1 variants *in vivo*, to guide the selection of appropriate treatment strategies for individual patients.

## Author contributions

EK and DF led the project and the manuscript production. MK and GH contributed to the manuscript preparation. All authors read and approved the final manuscript.

## Funding

This work was funded by the European Leukodystrophy Association (ELA 2018-014I2) and the Australian Government Medical Research Future Fund (Leukodystrophy Flagship – Massimo’s Mission; MRFF-ARLKO). EK was supported by an Australian Government Research Training Program (RTP) Scholarship.

## Conflict of interest

MK was employed by the company Boehringer Ingelheim Pharma GmbH & Co. KG.

The remaining authors declare that this literature review was produced in the absence of any commercial or financial relationships that could be construed as a potential conflict of interest.

## Publisher’s note

All claims expressed in this article are solely those of the authors and do not necessarily represent those of their affiliated organizations, or those of the publisher, the editors and the reviewers. Any product that may be evaluated in this article, or claim that may be made by its manufacturer, is not guaranteed or endorsed by the publisher.
